# Commentary: A settler physician perspective on Indigenous health, truth, and reconciliation

**Published:** 2018-07-27

**Authors:** Preston Smith

**Affiliations:** 1 Faculty of Medicine, University of Saskatchewan, Saskatchewan, Canada

Jaworsky’s brief commentary, A settler physician perspective on Indigenous health, truth and reconciliation^1^ is a superb, thoughtful, and powerful appeal for progress on the Truth and Reconciliation Commission’s Calls to Action, most specifically 18-24. The title and the introduction start in the right place with an immediate focus on the author’s identity as a settler physician. How we see ourselves determines how we see others and is an essential step towards recognizing that “First Nations, Inuit, and Metis peoples, as the original peoples of this country and as self-determining people peoples, have Treaty, constitutional and human rights that must be recognized and respected.”^2^

My own Canadian identity was comfortably rooted in an Irish heritage with immigration to Canada in the context of The Great Famine and English tyranny. Those oppressed ancestors became oppressors as participants in colonization and I and the institutions I represent continue to have a “role in the ongoing oppression of Indigenous peoples.”^1^ Beginning with that recognition I, medical schools, and all of Canadian healthcare can move on to the author’s next three action-oriented recommendations.

The author’s first recommendation is for education and amplifies the statement by the Honorable Senator Murray Sinclair, Chief Commissioner of Canada’s Truth and Reconciliation Commission “Education has gotten us into this mess, and education will get us out.” Jaworsky takes this even further by emphasizing that only by confronting and embracing truth will we move to reconciliation.

Jaworsky calls for medical educators to include in our teaching of the social determinants of health “a frank description of the historical realities of settler colonialism in Canada”.^1^ This must go far beyond a brief history of residential schools, start with education about the history and health of Indigenous peoples prior to the fifteenth century and chronicle the subsequent decline. A superb required reading might be “Clearing The Plains – Disease, Politics of Starvation, and the Loss of Aboriginal Life” by James Daschuk.

The CanMEDS Health Advocate role states “advocacy requires action”^3^ and Jaworsky clearly calls on us to practice what we teach in service provision, cultural safety, and engagement of Indigenous peoples in their healthcare. Jaworsky calls on all physicians but the TRC is even more explicit in calling on all “those who can effect change in the Canadian healthcare system”^2^ and medical schools, in particular, to lead and drive the change so that “we may finally find ourselves on a path towards reconciliation in healthcare.”^2^

Jaworsky exhorts us that own individual and collective actions can positively change the future of healthcare for Indigenous people. We need to do this for our Indigenous patients, communities, colleagues, residents and students. We need to create safe, respectful spaces to dialogue about racism and inequity in healthcare. And we must respect, support and listen to Indigenous people as they make their voices heard.

Change is a team sport!
